# A qualitative study of secondary distribution of HIV self-test kits by female sex workers in Kenya

**DOI:** 10.1371/journal.pone.0174629

**Published:** 2017-03-27

**Authors:** Suzanne Maman, Katherine R. Murray, Sue Napierala Mavedzenge, Lennah Oluoch, Florence Sijenje, Kawango Agot, Harsha Thirumurthy

**Affiliations:** 1 University of North Carolina at Chapel Hill, Gillings School of Global Public Health, Department of Health Behavior, Chapel Hill, North Carolina, United States of America; 2 FHI360, Durham, North Carolina, United States of America; 3 Research Triangle International, San Francisco, California, United States of America; 4 Impact Research and Development Organization, Kisumu, Kenya; 5 University of North Carolina at Chapel Hill, Gillings School of Global Public Health, Department of Health Policy and Management, Chapel Hill, North Carolina, United States of America; University of Toronto, CANADA

## Abstract

Promoting awareness of serostatus and frequent HIV testing is especially important among high risk populations such as female sex workers (FSW) and their sexual partners. HIV self-testing is an approach that is gaining ground in sub-Saharan Africa as a strategy to increase knowledge of HIV status and promote safer sexual decisions. However, little is known about self-test distribution strategies that are optimal for increasing testing access among hard-to-reach and high risk individuals. We conducted a qualitative study with 18 FSW who participated in a larger study that provided them with five oral fluid-based self-tests, training on how to use the tests, and encouragement to offer the self-tests to their sexual partners using their discretion. Women demonstrated agency in the strategies they used to introduce self-tests to their partners and to avoid conflict with partners. They carefully considered with whom to share self-tests, often assessing the possibility for negative reactions from partners as part of their decision making process. When women faced negative reactions from partners, they drew on strategies they had used before to avoid conflict and physical harm from partners, such as not responding to angry partners and forgoing payment to leave angry partners quickly. Some women also used self-tests to make more informed sexual decisions with their partners.

## Introduction

With nearly half the HIV-positive individuals in sub-Saharan Africa (SSA) being unaware of their serostatus,[1] there is a vital need for novel strategies to promote HIV testing. Increasing awareness of serostatus among those already infected is one of the UNAIDS “90-90-90” targets and represents the first step towards achieving the promise of treatment as prevention. While various community-based HIV testing and counseling (HTC) strategies have been shown to be effective in achieving high testing coverage,[2, 3] reaching key populations and other high-risk segments of the population remains a challenge.[3]

Promoting awareness of serostatus and frequent HIV testing is especially important among high risk populations such as female sex workers (FSW) and their sexual partners. FSW in SSA have HIV prevalence that often exceeds 50% and is 10–20 times higher than prevalence in the general population.[4, 5] Despite broad acknowledgement of their enhanced risk, however, FSW are largely neglected by HIV programmes in most countries in SSA[4, 5] and face a need for interventions that can promote frequent HIV testing, facilitate safer sexual behaviors, and ultimately reduce their risk of HIV infection. Male sexual partners of FSW, including clients and regular partners, represent another under-prioritized population that has limited engagement with HIV services, thereby contributing significantly to increased HIV transmission.[6, 7] Interventions aimed at promoting testing in these populations must overcome key barriers to testing such as stigma, fears about the confidentiality of venue-based testing, distance to testing sites, and opportunity costs.[8, 9]

HIV self-testing (HIVST) is an approach that is gaining ground in a number of countries worldwide.[10, 11] By providing an opportunity for individuals and even couples to learn their HIV status in a private setting and confidential manner, HIVST has considerable potential to increase access to HIV testing. A growing number of studies have documented high interest in and acceptability of HIVST in both high and low-income settings, as well as in the general population, healthcare workers, and key population groups such as FSW and men who have sex with men (MSM). An appealing aspect of HIVST is that acceptability has been high in groups that are typically less likely to seek testing using other HIV Testing Services (HTS) and that have higher risks of being HIV-infected.[10, 12–14] Some countries, such as Kenya, have already included regulated HIVST in their national HTS guidelines and several are considering scaling-up the availability of self-tests.[15, 16] Even as HIVST scale-up is considered, however, less is known about self-test distribution strategies that are optimal for promoting testing access among hard-to-reach and high risk individuals, as opposed to those already using facility- and community-based testing services. Whether certain strategies of distributing self-tests in the population will lead to a higher risk of social harms must also be explored further.

One promising strategy for expanding access to testing in hard-to-reach populations is ‘secondary distribution’ of self-tests, whereby an individual who is given multiple self-tests can distribute them to sexual partners or to others in their social network. A recent cohort study conducted by our team in Kenya demonstrated that 101 HIV-negative FSW who received five oral fluid-based self-tests along with general education on how to use the tests and counseling were successful in distributing these tests to their sexual partners, including clients.[17] FSW reported distributing self-tests to 301 male partners, among whom 298 (99%) were reported to have used the tests. In particular, 75% of FSW reported that their primary sexual partner accepted and used a self-test and 81% of FSW distributed ≥1 self-test to commercial sex clients. Among the FSW who reported distributing self-tests to their primary sexual partner, 83% reported that they used self-tests for couples testing. Importantly, among all sexual partners who accepted a self-test, FSW reported that 14% of the partners (n = 41) tested positive for HIV. Fewer FSW whose partners tested positive for HIV engaged in sexual intercourse with the partner after learning his diagnosis (7/41, 17%) as compared to 131/242 (54%) of women who reported that their partners tested HIV-negative. Among the 7 women who engaged in sex with an HIV-positive partner, all (7/7, 100%) reported using condoms with this partner as compared to only 91/131 (69%) of FSW who reported that they used condoms when engaging in intercourse with their partner after he tested HIV-negative. Adverse events, specifically intimate partner violence as a result of the use or distribution of HIVST, were reported by 2 of the 96 (2%) FSW at the three month follow-up visit. [17]

To further explore the potential risk and benefits of equipping FSW with multiple self-tests, this qualitative study explored factors shaping the decisions of FSW to offer self-tests to some of their partners, the strategies they used to introduce self-tests, and the reactions they received from their partners. We asked women to describe negative experiences they had with partners and other when distributing self-tests. We also explored sexual behaviors with partners after distribution of self-tests and the extent to which the self-testing experience led to couples testing and safer sexual decisions.

## Methods

### Background on parent study

This qualitative study was part of a larger cohort study in the Kenyan city of Kisumu conducted in January through May 2015 that assessed the acceptability and feasibility of secondary distribution of HIV self-tests as a strategy to promote partner testing and results disclosure, and facilitate safer sexual decision making.[17] HIV prevalence among FSW in Kisumu is estimated to be 56.5%[18] and recent data indicate there are 534 active FSW “hotspots” of commercial sex work, with an estimated 4,041 FSW.[19] In the larger study, described above and elsewhere,[17] 143 FSW were randomly selected for tracing by peer educators. Of these, 21 (15%) could not be located, 12 (8%) declined study participation, and nine (6%) were referred to study staff but did not meet study inclusion criteria, A total of 101 HIV-negative FSW were enrolled at a drop-in center that provides HIV prevention, care and treatment services to key populations. After providing informed consent, participants completed a baseline survey and then received instructions from study staff on how to use the OraQuik rapid HIV-1/2 antibody test (OraSure Technologies, Bethlehem, PA, USA). Prior to initiating enrollment, study staff underwent rigorous training that included how to explain self-test usage and what advice and suggestions to give to FSW around partner testing. Specifically, the study staff were trained in how to talk to FSW about the need to use discretion when deciding whether to offer a self-test to a primary partner or client. Staff talked to FSW about how to introduce the topic of HIV testing, gauge the openness of the partner to testing, and to assess potential risks before offering a self-test. Study staff demonstrated how to use the self-test, provided an instruction sheet with each self-test, and explained the “window period” during which the test would be unlikely to detect HIV antibodies. All women were given five self-tests and encouraged to distribute them to sexual partners and others. Ninety-six of the 101 FSW enrolled (95%) completed the three-month follow up assessment.

### Qualitative sub-study

#### Sampling and interview procedures

The qualitative study was conducted with a sub-sample of 18 of the 96 FSW who completed a three-month follow-up assessment in the larger study. Participants were purposively selected based on their responses to this assessment. We sampled women who reported a range of experiences sharing self-tests with different numbers and types of partners. Selection criteria used included: type of partner self-test was offered to (primary sexual partner, client, people who were not sex partners such as friends or other FSW) and response of that person (selected for both positive and negative experiences, as well as people who had offered the test to someone who refused to use it). Finally, we sampled all women who had particularly salient and unique experiences, for example a FSW who used a self-test kit to test the girlfriend of a client. Women were interviewed by local research assistants trained in qualitative research methods. Each interview was conducted in English, Kiswahili or Dholuo, as preferred by the participant. Interviews were audiotaped, transcribed and as needed translated into English for analysis. Interviewers used in-depth interview guides that outlined the major topics for each interview and included suggested probes. Interviews were divided into three sections. In the first section, women were asked about their current living situations, for example whom they were living with and how many other people were in the home, to gain a deeper understanding of their lives. In the second section, women were asked specific questions about each self-test that they used themselves and distributed to partners, clients or friends. For each self-test, women were asked why they selected that individual, how they introduced the self-test to them, where and how self-testing occurred, how the women and partners felt about the ease of self-testing, what reactions the partners, clients or friends had when they learned their results, and whether any sexual behaviors changed between the women and their sexual partners after they had tested. In the final section of the interview, women were asked whether they had any recommendations for how to improve the process of distributing self-tests to partners. The study received approval from the Office of Human Research Ethics at the University of North Carolina at Chapel Hill and the Ethical Review Committee at the Kenya Medical Research Institute. Eligible women who wished to participate provided written informed consent in their preferred language.

#### Data analysis

All data were analyzed using Atlas.ti version 7.2.[20] The team developed a code book for the deductive codes or codes that corresponded closely to the topics covered in each interview. The data were then coded using these deductive codes by a single coder. Another analyst generated code reports for each of the deductive codes and then constructed matrices to summarize each interview by the topic covered. The analyst reviewed the summary matrices against the original transcripts to insure that the summaries were accurate and complete. The summary matrix enabled us to compare patterns across the FSW. We used these matrices to identify emerging patterns within the topics which is the focus of our results below.

## Results

### Description of participants and the sexual partners who accepted self-tests from participants

The average age of the qualitative participants was 28.4 years and the range was 19–39 years. Eight of the women had a primary school education, eight had a secondary school education, and two reported a greater than secondary school education. All but one woman had children, and most women lived with their children and other relatives. Eleven of the women had never been married, and among the seven who had been married all were either separated or widowed from their husband. All women reported that their primary source of income was commercial sex work. Among the 18 FSW we interviewed, 12 reported having a primary sexual partner but only one of these 12 women lived with their primary partner. Table 1 summarizes the participants’ demographic characteristics.

**Table 1:** Demographic characteristics of participants (N = 18).

Age (years)	N(%)
18–24	4/18 (22.2)
25–29	8/18(44.4)
>30	6/18(33.3)
**Education**	
Some primary	8/18(44.4)
Some secondary	8/18 (44.4)
Secondary or greater	2/18 (11.1)
**Children**	
0	4/18 (22.2)
1	5/18 (27.8)
2–3	12/18 (66.7)
**Marital Status**	
Separated/divorced	7/18 (38.9)
Never married	11/18 (61.1)
**Currently has sexual partner**	12/18 (66.7)

The majority of women (n = 13) used all five of the test kits they were given, while three women used four of the kits and two women only used three of the five kits she received. The 18 women reported distributing self-tests to a total of 50 sexual partners, including 11 primary partners and 39 commercial sex partners (27 regular clients and 12 new clients). Among these 50 partners, seven were reported to have obtained an HIV-positive result (two were women’s primary partners, and five were clients), 40 men were reported to have obtained an HIV-negative result, and women were unsure of the result of the remaining three partners. Table 2 summarizes key outcomes regarding self-test distribution to partners by women, which will be described in more detail in the sections below.

**Table 2:** Experiences of female sex workers who received multiple self-tests (N = 18).

Number and type of partners who accepted and used self-tests	N(%)
Primary partner	11/12 (91.7)
Regular client	27
New client	12
**Number and type of men who tested HIV-positive**	
Primary partner	2/11(18.2)
Regular client	3/27 (11.1)
New client	2/12 (16.7)
**Number of women who tested with partner**	
With primary partner	11/18 (61.1)
With regular client	8/18 (44.4)
With new client	6/18 (33.3)
**Number of women who were verbally/physically assaulted**	
By primary partner	0/18 (0)
By regular client	2/18 (11.1)
By new client	0/18 (0)

### The self-testing experience with male partners

#### Self-testing with primary partners

Eleven of the twelve women who reported having a primary partner said they distributed a self-test to their primary partner. The one woman who did not said it was due to the fact that her partner traveled for work and she had not seen him since she received self-tests from study staff. Two of the women had primary partners who tested HIV-positive and the remaining nine primary partners tested HIV-negative. Women said they gave the self-tests to their primary partners because they felt a greater level of commitment to these partners, often due to the financial support they provided to her and her family. Participants also indicated a greater level of intimacy and love with these partners, and wanted to know the status of these partners as this 22 year-old woman describes: “*Because he is the person I love and I trust him*. *No wonder I decided to start with him first*. *I wanted him to know his status*…*I wanted him to have the knowledge*.*”*

In the case of primary partners, all the 11 women reported also using a self-test at the same time and testing as a couple. In general, women had little trouble convincing primary partners to test for HIV. In two of the cases where the primary partner did not initially agree to use the self-tests, women said that eventually they convinced the partners either by withholding sex with him or by talking to him about the importance of knowing their status. This 26 year old woman describes how she talked to her partner when he initially resisted the idea of testing:

*At first, he told me that, I was, what, I was playing around with him at first. He told me that how come I have such things [self-test kits], how come that I was very eager to know his status. So I told him this is me and this is you….okay, this man, I gave him this kit because he is always loving, to that boy of mine, so this is the man that I trusted, and more so, I always meet with him, no wonder I gave him the kits. And I told him, I have some kits. The first question he asked is, where did I get the kit from? So, I explained. At first, he did not want me to test him with the kit, because he thought, I was only going to test him and not me. So I told him that we were free. I used the kit and we tested each other, and it turned negative*.

Women generally described the HIV self-testing process with their primary partner as a positive experience. Even though they often said their partner was anxious about the testing and that there was some tension while they waited the 20 minutes for the test results, in many cases introducing the self-test to the primary partner and waiting for the results provided the couple with an opportunity to talk about sexual risk and how to keep themselves healthy. This 20 year old woman described the conversation she had with her partner while they waited for the results:

***So what was it like testing together?** The feeling was good, I felt I like he trusted me and he was free with me, yes. The results came out negative and we felt good about it and we promised to protect each other just like that. I felt good when I was clean [HIV-free], yes and our baby was safe too. He felt happy, he was just smiling and then he said I told you to trust me, I was fine, why bring the kit around? Next, I told him, yes this is a new kit, you do just have to believe in it, let us just go and test it in the hospital again and then he told me it is fine, I will just do it because I am now sure of myself so much, yes. **Thank you, so did you talk about the results with each other, you and him?** Yes, we saw the results both of us, so we talked about it, and to maintain the results, we had to play safe. I told him you don’t have to ‘hanya’ [go out with other girls] outside*…

#### Self-testing with commercial sex clients

Sixteen of the 18 women interviewed reported that they had distributed a self-test to at least one client, with most women distributing self-tests to two or more clients. A total of 39 clients were reached through secondary distribution, including 27 clients that women saw on a regular basis and 12 new clients. Five of these clients tested HIV-positive, 31 tested HIV-negative, and women were unsure about the results of the remaining three clients (usually because they were not present when the client used the self-test). Among the 39 clients, 14 tested with the woman as a couple and 25 tested alone but often in the presence of the woman. The 14 cases of couple testing with clients were concentrated among eight of the FSW. Two of these FSW tested as a couple with one client each, and six tested with two clients each. Women who distributed self-tests to new clients said they chose to do so because they were excited to use the tests with clients, and these were the first clients they encountered after they received the kits. In a few cases, they also said that they distributed the tests to the new clients whom they judged to be mature and unlikely to react negatively to the suggestion of an HIV test. Women who distributed self-tests to their regular clients gave a number of reasons for choosing to do so. In some cases, they selected clients who they saw on a more regular basis because they knew these individuals and could gauge how they would react to the suggestion of HIV testing. These clients often supported the women financially, and as a result they felt some commitment to those partners as this 22 year old woman described:

*I went to his place, after going to his place, I cooked for him, we ate, it then reached night time, at night before we started our things [Chuckles], I started telling him stories. I pretended that I was telling him a story about my friend, how he, you know when I am with him, I tell him he is the only one that I love and it’s only him [Laughter]. That there is no one else [Chuckles], I told him it is good that when people are staying together, they should know their status. So, he asked me what I am trying to say, he told me to be open with him so I can tell him what I am trying to say. Then I told him that I have a thing, if someone wants to know their status, and in case he wants to know his status, I can teach them how to look at it and know their status, he didn’t believe at first. I saw him change and he asked me why am I bothering him, I told him it’s because I loved him and that was why I was bothering him, I just want him to know his status, and I told him that I am negative, and we can go with him to the nearest VCT so that he can see that I am negative. Then he agreed, and I gave him that kit, mmm. **Okay, thank you and how did you decide that it was him that you would use that kit with, that client?** I decided it was him, because he is also that person who is important to me, not important in that I love him but because he gives me good money*.

Fourteen of the 16 women who distributed self-tests to their commercial sex clients reported neutral or positive reactions of these clients during and after use of the self-test. In a few cases, the women had tested previously with the clients so this self-testing experience was an extension of their prior testing experience together. In other cases, the men were very open and interested in using the self-test and learning their status, and the testing experience led to dialogue about risk and reinforced the importance of having protected sex for both of them as this 21 year old woman describes:

**How did he react when you told him that you had something and you wanted you and him to test how did he react?** I just saw him happy. **What were you doing while you were waiting for the results?** We were conversing. I asked him if we test and he is found to be having the disease. And he also asked me if am found to be having [the disease] what will we do. And I told him not to fear it is something that is there...Eeh I told him that we could get drugs and continue with it. Drugs that suppresses the virus. **And how was the result?** The result I didn’t find him with it, he was not having it (means HIV). **And how did he react to this?** He was happy. **Did you talk about your results together**? Yes. He encouraged me that we should continue testing…**Thank you so when you had tested and you got the result did anything change as a result of taking the HIV self-test?** We were happy because we knew our status. **And did you have sex after the test? Did you use condoms?** We have been using condoms I told him we continue using condoms.”

Two of the women reported challenging experiences following distribution of self-tests. One of these two women reported experiencing both sexual abuse and verbal abuse (sexual abuse from one client and verbal abuse from two clients), while the other woman experienced verbal abuse from one client. The verbal abuse by clients occurred when the two women suggested HIV testing. The clients berated the women, asking them why they suggested testing, believing that this is something only a wife could suggest. Among the three verbally abusive clients, two eventually tested in the presence of the women. One of these clients tested positive and the other did not show her his results, but she assumed he was positive based on his reaction after testing. In the case of these two clients, the women left the clients after they tested, without having sex. In the case of the third verbally abusive client, the man did not test in front of the woman. After having sex with the woman, this client took the test kit with him when he left and said he would contact her with the results. This was a client the woman saw every month and regularly had unprotected sex with, thus she wanted to know his status. She had not heard from him by the time of the interview and therefore did not know his HIV status. The 22 year old woman who reported being sexually assaulted by one of her clients described her experience below:

***How did this person react when you brought up the topic of HIV self-testing*?** *He took it negatively…he changed and told me to keep quiet*, *and he pushed me immediately on the bed*, *and told me those words that*, *he has to sleep with me without a condom*, *since there is no where I can take him*. *I couldn’t run away coz I was also drunk and you also know the clothes that we always wear*. *They are short*, *so you know when someone has thrown you on the bed; they just pull it like this*, *[demonstrating with arms] and remove it and do their business*. *He was a big person; I couldn’t match his strength with mine…*.*I was just crying as I didn’t know his status and he had already [previously] slept with me without a CD [meaning condom] so I was asking myself*, *if he has given me [infected me with HIV] then ‘whose visitor was I going to be’ [meaning who was going to welcome her]*. ***So*, *you had sex before the results*?** *We had sex before results*. *He tested alone*. *His results came out negative*. *He just took it well but didn’t even thank me*. *After the result was out*, *it found when he had already abused me and slept with me without using a condom*, *and then*, *it found that he had already paid me*, *immediately after knowing his status*, *I got up and went to take a bath*, *then I put on my clothes*, *I went and left him there*.

### Distribution of self-tests to non-partners

Six of the women distributed self-tests to other individuals in their social networks. Two women gave three of their kits to friends or sisters, while the remaining four women distributed just one of their five kits to a friend or relative. Most women who distributed to a friend or sister said they decided to do this because they felt that this person was at risk for HIV, and they wanted them to know their status. This 26 year old woman describes sharing a self-test with her neighbor because her neighbor wanted to test but feared going to a clinic to get a test:

*There are people who don’t test, they fear! Mh! **Your neighbor also fears?** Yes. I told her that there were some things that are there that I came with from Impact [NGO conducting the study] where I usually go and I wanted to give her one so that she could test herself. That she could even test alone secretly. She told me to give her. After I gave her she went with it to her house and she tested herself. **Did she tell you her results?** Yes. It was negative*.

Another woman used one of the self-test kits in a way that it was not intended to be used. This woman initially self-tested with one of her regular clients, and they both tested negative. The client then asked her to come the next day to his house and pretend to be his sister so that she could test his other girlfriend. The woman went to his house the next day and did as he asked. The man’s other girlfriend was a young female student in Standard 8 (equivalent to 8^th^ grade in US). The young woman tested HIV-positive. She said that her client chased the girl from his house as he did this ‘he was somebody who looked like he could kill her.’ The woman said she tried to follow-up with the girl after she was chased from his home, but she has not been able to make contact with her.

### Distribution of self-testing led to more informed sexual decision making

One of the goals of the intervention was to provide women with a tool to learn their sexual partners’ HIV status so they could make more informed decisions about sexual behaviors. The narratives of women’s testing experiences provide insight into how women used the self-tests to both gauge their risk and to engage in conversations about HIV and HIV risk with partners.

#### Sexual risk with primary partners

Among the eleven women who reported that they distributed the self-test kits to their primary partners, eight reported that they did not use condoms regularly with their partner prior to self-testing, and three reported they did regularly use condoms with their primary partner. One woman who used condoms prior to testing with her partner said that she and her partner decided to stop using condoms after they both tested negative. She told the interviewer, *“There was no need of us to use condoms*. *We put aside condoms*.*” (FSW*, *35 years*). The other women did not report a change in condom usage with their primary partner after testing. Two of the women whose partners tested positive reported that they were no longer having sex with that partner. Both of these women reported having unprotected sex with their HIV-positive primary partner prior to learning his status through the HIV self-test. These relationships did not end in conflict. Rather, the partners either lost contact or explicitly decided not to continue to see each other as described by this 26 year old woman:

*No, I did not coerce him, I only told him that it is voluntary and that if he agreed, yes, then he agreed nicely. **So, what was this person’s reaction when you brought up the topic of HIV self testing?** He did not take it badly, he just tested as he smiled and later when he found out that he was positive, he said that he must again test at the hospital to confirm and I said yes, it is okay and am ready, so the next day we again went to the hospital and found the same result. **Thank you, so what happened next?** After that we just had an argument that he was still going to give me the baby’s support because for me now there was nothing I can say. Yes, am now the one who got shocked. Now, we decided that he should just support the baby. Between us, I am the one who decided that I can’t do it anymore, I felt frightened out of shock. **Thank you and did you talk about the results together with him after realizing he was positive**. No, we did not talk; we just left that we will go first to the hospital and confirm our results again, so we found the result at the HTC [HIV testing and counseling] it is when we talked. **So did anything change as a result of taking the test?** Yes. After going to the hospital for confirmatory testing, things must now change because I don’t know how he lives and I would also be infected and the best thing is I knew his status on time and he thanked me for saving his life too. In terms of our relationship, we still talk to each other but only for the sake of our baby to assist with its upbringing*.

#### Sexual risk with clients

Distribution of self-tests to clients made it possible for participants to reduce their risk of HIV acquisition. There were five clients who accepted a self-test and obtained a positive result; three of whom were regular clients. In four of these cases, participants did not have any sexual intercourse with the client after self-testing, and reported that the relationships had ended as a result. In the remaining case, the woman continued to have sexual intercourse with the HIV-positive client and continued to use condoms with the client.

At the same time, regular condom use prior to self-testing was reported by 25 of 27 regular clients who accepted self-tests from participants, and all but one woman reported continuing to use a condom with these clients following self-testing. Only one 28 year old woman reported agreeing to have unprotected sex with two clients as a result of the client obtaining an HIV-negative test result. One of these clients was a new client, while the other was a client with whom she had been having sex with condoms prior to self-testing, as she describes:

***Did anything change as a result of taking the test*?** *Yes. The nature of love changed a bit. Because the nature of love changed we knew our status. You know we stay far from each other. Yes it changed the nature of love because even if he wanted to use a condom for that day and he finds out that am clean [meaning HIV-negative] and he is clean too, he concluded there is no need for us to use the condom*. ***Thank you. Did you engage in sex after testing*?** *Yes we had sex*. ***Did you use condom*?** *No we did not use*.

In four cases, women encouraged clients to accept testing by telling them that they would agree to have unprotected sex with them only if they used the self-test and showed that they were HIV-negative. In all of these cases, the client tested HIV-negative but the women deliberately avoided having unprotected sex with these clients by telling them they still had to go for confirmatory test before they could be sure of their HIV status. This was clearly a strategy that the women described using to avoid unprotected sex with the clients. In one of these cases, the man provided the woman with a larger sum of money, 1,000 Ksh (equivalent to US$10) if she agreed to have sex with him without a condom. The woman said she would only agree to unprotected sex if he agreed to test and his results were negative. The man tested, and his results were negative, and the woman still insisted on condom use. The two had sex with a condom, and this led to an argument, which ended by him giving her only half the money they agreed upon up front.

## Discussion

The purpose of this qualitative study was to explore the experiences of 18 FSW in Kenya who participated in a larger study to assess whether provision of multiple HIV self-tests to women can help promote male partner testing through ‘secondary distribution’ and also facilitate more informed sexual decision making by the women. While not meant to be fully representative of the experiences of all FSW who participated in the larger study, the experiences described by these 18 women illustrate the ways in which this strategy can increase access to testing for male partners and also influence women’s sexual decision making. They also provide further insight on less frequent cases in which women have negative experiences with the introduction of self-tests to their partners. The 18 FSW distributed self-tests to 50 male partners, including 11 primary partners and 39 commercial sex partners. Both women and the male partners expressed high enthusiasm for self-tests primarily because they could learn their status in a private setting.

Women demonstrated agency both in the strategies they used to introduce self-tests to their partners and in the strategies they used to avoid conflict with partners. Women carefully considered with whom to share self-tests, often assessing the possibility for negative reactions from partners as part of their decision making process. Women generally chose men with whom they felt more committed, often because these primary or commercial partners financially supported the women and their children. Women used creative approaches to encourage initially reluctant partners to accept and use the self-test, for example, withholding sex from partners unless they tested. As with any instance of HIV testing, in the presence of a counselor or not, women reported that they and their partners were sometimes nervous as they awaited the test results. But in nearly all instances, women reported that they were pleased to have access to multiple self-tests and be able to learn the HIV status of their sexual partners. They also reported that their partners were generally glad to have the ability to learn their own status easily. In many cases, the access to multiple self-tests allowed to women to test with their partner at the same time, as a couple. Overall, for many women and their partners, the access to multiple self-tests also gave rise to discussions about sexual risk reduction strategies.

Secondary distribution of HIV self-tests by FSW was not without risks. When women faced negative reactions from partners, they drew on strategies they had used previously to avoid conflict and physical harm from partners, such as not responding to angry partners and forgoing intercourse to leave angry partners quickly. These narratives highlight the fact that FSW often face violence and other potential harm from partners [21] and they have systems and strategies that they use to keep themselves safe in light of these risks. In the larger cohort study, 2 (2%) of the 101 FSW participants reported experiencing physical or sexual abuse resulting from self-test distribution. This qualitative study purposively sampled one of the two participants who reported experiencing such abuse in the quantitative phase of the study, and obtained additional evidence of verbal abuse from another participant. While there are few comparable studies of partner testing interventions among FSW, the proportion of FSW participants who reported negative experiences with partners following self-testing is comparable to or lower than what has been reported in other studies of interventions that sought to promote male partner testing among women in the general populations.[22, 23]

Despite the negative experiences reported by two women, it is also notable that at the end of the interview when asked what the program could do to improve the distribution of self-test kits to clients, the woman who had negative experiences with three separate partners continued to express enthusiasm for the intervention. She told the interviewer she would like to have more kits to distribute to other partners. Nonetheless, as HIVST scale-up begins to occur, there is a need to both monitor and address the potential for negative outcomes among vulnerable groups that use self-tests themselves or with their sexual partners. Greater education about the need to consider the risk of negative reactions when offering tests to current or potential sexual partners as well as provision of support services is essential. At the same time, it is noteworthy that the experiences of FSW who participated in this study by and large suggest that they were not at greater harm due to the secondary distribution of self-tests than they would have been if they promoted facility-based testing with their partners.

This study also explored how the distribution of self-tests to partners affected sexual behavior. Overall, these qualitative findings were largely consistent with quantitative data obtained from all participants in the larger study.[17] The self-testing experience often resulted in open discussion between partners about HIV risk, the importance of prevention and the value of knowing one’s status, particularly when the testing was done as a couple as was the case in a substantial proportion of the testing experiences of commercial sex clients of women who participated in the qualitative interviews. It was apparent from the patterns of couple testing with clients, that some of the FSW were more successful than others are promoting couple testing. The 14 cases of couple testing with clients were concentrated among eight of the FSW, and six of these FSW had two commercial clients who tested with them as a couple. There were several women who described a reduction in sexual risk as a result of the self-testing experience, and this primarily came in the form of ending relationships with HIV-positive partners or partners whom women were unsure of their status. Given that FSW often have clients willing to pay a higher price for unprotected sex,[24] the availability of self-tests may be a useful tool for those FSW who do engage in unprotected sex with some of their clients to reduce their risk for HIV. At the same time, counseling FSW to continue to use condoms with their partners regardless of the HIV test results remains important, as one woman reported that she stopped using condoms with two of her partners after they self-tested HIV-negative for HIV. Despite the very different study setting and population, these findings are similar to those obtained from a similar self-testing intervention that was implemented among MSM in the US. In particular, the pilot studies conducted with high risk MSM who were given multiple self-tests have shown that this intervention may influence sexual behavior by facilitating more informed, safer sexual decision making.[25–27]. Despite these potential benefits, the risks associated with the use of self-tests for sexual decision-making must also be recognized, particularly if individuals do not full understand the 3-month window period. For this reason, WHO has recommended against the use of self-tests for serosorting and sexual decision-making in population groups with high HIV-incidence such as sex workers [14]. Programs that promote HIVST need to continue to raise awareness of the three-month window period and the need for confirmatory testing.

While women who participated in this study received multiple self-tests as part of the intervention being assessed, as HIV self-tests become more widely available and affordable, it is inevitable that some individuals will acquire multiple self-tests in other ways and consider using them to learn their partners’ status. Thus, this study provides insights on the wider implications of scaling-up HIVST by showing that women who gain access to multiple self-tests are capable of using discretion when deciding whether to offer self-tests to their sexual partners. Even in settings with high levels of IPV such as Kenya,[28] this self-selection into choosing whether to offer self-tests to partners can explain why the risk of violence or other adverse events may be low when women have access to multiple self-tests. The women who were enrolled in this study self-identified as FSW, thus the study cannot be generalized to other high risk women who may engage in transactional sex but do not self-identify as FSW.

This study did not include interviews with any partners of FSW. As a result it was not possible to glean additional information on the characteristics of men who did and did not accept self-tests from the FSW, or to verify whether the women’s reported reactions of their male partners were accurate. In a study currently being planned in the same setting, qualitative work will explore men’s views about interventions that involve distribution of self-tests by FSW. In addition, while all women who received self-tests were informed that reactive test results must be confirmed by further HIV testing in the presence of a trained provider, we were unable to establish whether the men who accepted a self-test from the FSW and obtained an HIV-positive result sought confirmatory testing afterwards. In some cases, FSW did not know whether a client sought confirmatory testing because they did not have further contact with the client. Nonetheless, further research is needed to estimate rates of confirmatory testing as well as linkage to care after HIVST and interventions to promote confirmatory testing may be needed in some cases. While HIVST has been shown to be highly sensitive and specific, user errors in performing or misinterpreting the results are not uncommon and as a result the ongoing need for continued messaging around the importance of confirmatory testing is critical, as noted by the WHO [14].

## Conclusions

This study highlights the value of secondary distribution of self-tests as a strategy to achieve *multiple* HIV prevention objectives among FSW and their sexual partners, including promotion of serostatus knowledge and reduction of sexual risk. The vast majority of women who were given multiple self-tests and interviewed qualitatively described positive experiences engaging their primary partners and clients with testing and leveraging the testing experience to also initiate conversations and make decisions about sexual risk reduction. Women demonstrated considerable agency in the strategies they used to introduce self-tests to their partners and to avoid conflict with partners. Despite overall positive experiences among the women, there were a couple of women who reported negative experiences with partners following introduction of the HIVST. The findings underscore the importance of making women aware of the potential for negative reactions from partners, and providing support services to women who need them following distribution of HIVST.

## References

[1] UNAIDS. 90-90-90: An ambitious treatment target to help end the AIDS epidemic. Geneva: UNAIDS, 2014.

[2] Suthar AB, Ford N, Bachanas PJ, Wong VJ, Rajan JS, Saltzman AK, et al Towards universal voluntary HIV testing and counselling: a systematic review and meta-analysis of community-based approaches. PLoS Med. 2013;10(8):e1001496 Epub 2013/08/24. 10.1371/journal.pmed.1001496 23966838 PMC3742447

[3] Sharma M, Ying R, Tarr G, Barnabas R. Systematic review and meta-analysis of community and facility-based HIV testing to address linkage to care gaps in sub-Saharan Africa. Nature. 2015;528(7580):S77–85. 10.1038/nature16044 26633769 PMC4778960

[4] WHO. Preventing HIV in Sex Work Settings in sub Saharan Africa. Geneva: World Health Organization, 2011.

[5] Beyrer C, Crago AL, Bekker LG, Butler J, Shannon K, Kerrigan D, et al An action agenda for HIV and sex workers. Lancet. 2015;385(9964):287–301. 10.1016/S0140-6736(14)60933-8 25059950 PMC4302059

[6] Alary M, Lowndes CM. The central role of clients of female sex workers in the dynamics of heterosexual HIV transmission in sub-Saharan Africa. AIDS. 2004;18(6):945–7. 15060443 10.1097/00002030-200404090-00013

[7] Voeten HA, Egesah OB, Ondiege MY, Varkevisser CM, Habbema JD. Clients of female sex workers in Nyanza province, Kenya: a core group in STD/HIV transmission. Sex Transm Dis. 2002;29(8):444–52. 12172528 10.1097/00007435-200208000-00003

[8] Musheke M, Ntalasha H, Gari S, McKenzie O, Bond V, Martin-Hilber A, et al A systematic review of qualitative findings on factors enabling and deterring uptake of HIV testing in Sub-Saharan Africa. BMC Public Health. 2013;13:220 Epub 2013/03/19. 10.1186/1471-2458-13-220 23497196 PMC3610106

[9] Shand T, Thomson-de Boor H, van den Berg W, Peacock D, Pascoe L. The HIV blind spot: men and HIV testing, treatment and care in sub-Saharan Africa. IDS Bulletin. 2014;45(1):53–60.

[10] Napierala Mavedzenge S, Baggaley R, Corbett EL. A review of self-testing for HIV: research and policy priorities in a new era of HIV prevention. Clinical infectious diseases: an official publication of the Infectious Diseases Society of America. 2013;57(1):126–38. 23487385 10.1093/cid/cit156 PMC3669524

[11] Johnson C, Baggaley R, Forsythe S, van Rooyen H, Ford N, Napierala Mavedzenge S, et al Realizing the potential for HIV self-testing. AIDS Behav. 2014;18 Suppl 4:S391–5. 24986599 10.1007/s10461-014-0832-x

[12] Figueroa C, Johnson C, Verster A, Baggaley R. Attitudes and Acceptability on HIV Self-testing Among Key Populations: A Literature Review. AIDS Behav. 2015;19(11):1949–65. 10.1007/s10461-015-1097-8 26054390 PMC4598350

[13] Choko AT, Desmond N, Webb EL, Chavula K, Napierala-Mavedzenge S, Gaydos CA, et al The Uptake and Accuracy of Oral Kits for HIV Self-Testing in High HIV Prevalence Setting: A Cross-Sectional Feasibility Study in Blantyre, Malawi. PLoS medicine. 2011;8(10):e1001102 Epub 2011/10/13. 10.1371/journal.pmed.1001102 21990966 PMC3186813

[14] WHO. Guidelines on HIV self-testing and partner notification: supplement to consolidated guidelines on HIV testing services. Geneva: 2016. 27977094

[15] World Health Organization. Consolidated Guidelines on HIV Testing Services. Geneva, Switzerland: World Health Organization, 2015.

[16] National AIDS and STI Control Programme. Guidelines for HIV Testing Services in Kenya. Nairobi: National AIDS Control Programme, 2015.

[17] Thirumurthy H, Masters S, Napierala Mavedzenge S, Maman S, Omanga E, Agot A. Promoting male partner HIV testing and safer sexual decision making through secondary distribution of self-tests by HIV-negative female sex workers and women receiving antenatal and post-partum care in Kenya: a cohort study. The Lancet HIV. 2016;3(6):e266–e74. 10.1016/S2352-3018(16)00041-2 27240789 PMC5488644

[18] Vandenhoudt HM, Langat L, Menten J, Odongo F, Oswago S, Luttah G, et al Prevalence of HIV and other sexually transmitted infections among female sex workers in Kisumu, western Kenya, 1997 and 2008. PloS one. 2013;8(1):e54953 10.1371/journal.pone.0054953 23372801 PMC3553007

[19] Odek WO, Githuka GN, Avery L, Njoroge PK, Kasonde L, Gorgens M, et al Estimating the size of the female sex worker population in Kenya to inform HIV prevention programming. PLOS ONE. 2014;9(3):e89180 10.1371/journal.pone.0089180 24595029 PMC3940432

[20] ATLAS.ti. Berlin: Scientific Software Development; 2016.

[21] Shannon K, Strathdee SA, Goldenberg SM, Duff P, Mwangi P, Rusakova M, et al Global epidemiology of HIV among female sex workers: influence of structural determinants. Lancet. 2015;385(9962):55–71. 10.1016/S0140-6736(14)60931-4 25059947 PMC4297548

[22] Osoti AO, John-Stewart G, Kiarie J, Richardson B, Kinuthia J, Krakowiak D, et al Home visits during pregnancy enhance male partner HIV counselling and testing in Kenya: a randomized clinical trial. AIDS. 2014;28(1):95–103. 23942059 10.1097/QAD.0000000000000023 PMC4337402

[23] Rosenberg NE, Mtande TK, Saidi F, Stanley C, Jere E, Paile L, et al Recruiting male partners for couple HIV testing and counselling in Malawi's option B+ programme: an unblinded randomised controlled trial. The lancet HIV. 2015;2(11):e483–91. 10.1016/S2352-3018(15)00182-4 26520928 PMC4656790

[24] Jakubowski A, Omanga E, Agot K, Thirumurthy H. Large price premiums for unprotected sex among female sex workers in Kenya: a potential challenge for behavioral HIV prevention interventions. JAIDS Journal of Acquired Immune Deficiency Syndromes. 2015;in press. 10.1097/QAI.0000000000000929 27097367

[25] Balan IC, Carballo-Dieguez A, Frasca T, Dolezal C, Ibitoye M. The Impact of Rapid HIV Home Test Use with Sexual Partners on Subsequent Sexual Behavior Among Men Who have Sex with Men. AIDS Behav. 2013. Epub 2013/05/10. 10.1007/s10461-013-0497-x PMC3815512 23657758

[26] Carballo-Dieguez A, Frasca T, Dolezal C, Balan I. Will Gay and Bisexually Active Men at High Risk of Infection Use Over-the-Counter Rapid HIV Tests to Screen Sexual Partners? Journal of sex research. 2012. Epub 2012/02/02. 10.1080/00224499.2011.647117 PMC3600862 22293029

[27] Carballo-Dieguez A, Frasca T, Balan I, Ibitoye M, Dolezal C. Use of a rapid HIV home test prevents HIV exposure in a high risk sample of men who have sex with men. AIDS Behav. 2012;16(7):1753–60. Epub 2012/08/16. 10.1007/s10461-012-0274-2 22893194 PMC3458207

[28] Kenya National Bureau of Statistics. Kenya Demographic and Health Survey 2008–09. Nairobi: Kenya National Bureau of Statistics, 2010.

